# Claudin 1 promotes blood-borne metastasis by modulating the Src/Ezrin signaling pathway in high-CLDN1 triple-negative breast cancer model

**DOI:** 10.1080/23723556.2026.2749929

**Published:** 2026-10-23

**Authors:** Huiping Yang, Hong Lu, Wei Wang, Junchao Ma, Ling Xiong, Yong Zou, Jianjiang Fu

**Affiliations:** a Department of Pharmacology, School of Pharmacy, Jiangxi University of Chinese Medicine, Nanchang, Jiangxi Province, China; b Network and Information Technology Center, Jiangxi University of Chinese Medicine, Nanchang, Jiangxi Province, China; c Tumor Therapeutics Research Laboratory, Jiangxi Province Key Laboratory for Diagnosis, Treatment, and Rehabilitation of Cancer in Chinese Medicine, Nanchang, Jiangxi Province, China

**Keywords:** Claudin-1, metastasis, circulating tumor cells, migration, triple-negative breast cancer

## Abstract

This study aimed to elucidate the role and mechanism of CLDN1 in regulating migration and metastasis in triple‑negative breast cancer (TNBC). *In vivo*, an orthotopic TNBC model was used to evaluate circulating tumor cells and lung metastasis; *in vitro*, cell migration, F‑actin organization, and protein expression were examined in cultured TNBC cells. CLDN1 was knocked down or overexpressed via lentiviral constructs, and Ezrin was silenced by siRNA in CLDN1‑overexpressing cells. Pharmacological targeting of CLDN1 with PDS‑0330 specifically reduced migration in MDA‑MB‑468 cells but not in MDA‑MB‑231 cells; knock-down and knock‑in experiments further confirmed that CLDN1 critically determines the migratory and metastatic capacity of TNBC cells with high CLDN1 expression. Analysis of Ezrin and its interaction with podocalyxin revealed that Ezrin significantly contributes to CLDN1‑promoted migration, and our data also indicated that CLDN1 facilitates Src signaling activation. Collectively, these findings demonstrate that in TNBC with high CLDN1 expression, CLDN1 plays a pivotal role in regulating migration and hematogenous metastasis, likely through upregulating the Src/Ezrin pathway.

## Introduction

The triple-negative breast cancer (TNBC) subtype lacks estrogen receptor (ER), progesterone receptor (PR), and human epidermal growth factor receptor 2 (HER-2) expression, accounting for 10%–20% of all breast cancers.^1^ ^,^ ^2^ TNBC demonstrates more high-risk factors, such as advanced stage, and greater lymph node involvement than other breast cancer types. It is also characterized by high invasiveness and metastasis, making it the most aggressive breast cancer subtype. The development of precise treatment strategies for TNBC lags that for other subtypes due to insufficient understanding of its biology.^3^ In recent years, efforts to explore targeted strategies for TNBC have shifted from a focus on classic biomarkers characterized by “negative” expression to an emphasis on identifying “positive” biomarkers that reflect this subtype's biological characteristics, including transcription, microenvironment, and metabolism.^4-6^ By comprehensive multi-omics profiling, Jiang et al. classified 465 Chinese TNBC cases into four transcriptome-based subtypes (luminal androgen receptor (LAR), immunomodulatory (IM), basal-like immune-suppressed (BLIS), and mesenchymal-like (MES)), each with distinct genomic alterations and potential therapeutic targets.^7^

Claudin-1 (CLDN1) is a crucial member of the tight junction protein family and is a primary component of tight junctions.^8^ Research has demonstrated that CLDN1 is predominantly localized at the cell surface of epithelial cells^9^ and fibroblasts^8^ under normal conditions. However, CLDN1 is aberrantly expressed under pathological conditions;^10^ in particular, it plays a significant role in the malignant progression of cancer by mediating epithelial–mesenchymal transition (EMT),^11^ participating in cellular migration and invasion,^12^ and promoting cancer stemness and cell chemoresistance.^13^ CLDN1 expression exhibits significant heterogeneity across breast cancer subtypes. In luminal A and B subtypes, which are characterized by ER positivity, CLDN1 expression is generally low, with only approximately 5% of cases showing positive staining. In contrast, ER-negative breast cancers display markedly higher CLDN1 expression, with positivity rates reaching 39%. Within the ER-negative subgroup, the basal-like breast cancer (BLBC) subtype shows the highest CLDN1 expression levels.^14^ A separate study of 86 basal-like cancers further confirmed that 63% exhibited high CLDN1 expression.^15^ As the most aggressive form of ER-negative breast cancer, TNBC largely overlaps with the basal-like subtype and similarly demonstrates elevated CLDN1 expression, which has been associated with adverse prognostic features including invasion and metastasis.^16^ These studies suggest that, in most TNBC cases, a pattern characterized by high expression and abnormal localization is positively correlated with disease progression. These findings suggest that abnormal CLDN1 expression patterns render its potential role in the progression of CLDN1-high TNBC and may serve as a “positive” biomarker reflecting this disease's biological characteristics.

Given the findings, we propose that CLDN1 may play a pivotal role in driving the malignant phenotype of TNBC and may serve as a therapeutic target. However, the exact role of this protein in TNBC progression and its regulatory mechanisms remains unclear. Therefore, this study was conducted to: (1) compare the migration and metastasis capabilities of MDA-MB-231 (low CLDN1 expression) and MDA-MB-468 (high CLDN1 expression) TNBC cells; and (2) investigate the mechanisms by which CLDN1 facilitates metastasis in cells with high CLDN1 expression.

## Materials and methods

### Materials

AZD0530 (Cat. No. HY-10234), a tyrosine kinase inhibitor against Src family kinases, and PDS-0330 (Cat. No. HY-153833), an inhibitor of CLDN1, were purchased from MedChemExpress (Shanghai, China). The RNeasy mini and OneStep Ahead PCR kits were obtained from QIAGEN (USA). The Oris™ cell migration system was purchased from Platypus Technologies (USA). AlexaFluor488® Phalloidin was purchased from ThermoFisher Scientific (USA). LV‑CLDN1 shRNA (CLDN1 Human shRNA Lentiviral Particle, Locus ID 9076) and LV-CLDN1 (CLDN1 Human Tagged ORF Clone Lentiviral Particle, NM_021101) are products of OriGene Technologies (USA). Ezrin siRNA (SignalSilence® Ezrin siRNA I, Cell Signaling Technology, Inc.) and SignalSilence® Control siRNA were purchased from Cell Signaling Technology (USA). CLDN1 monoclonal antibody and phospho-CLDN1 Polyclonal Antibody were purchased from Invitrogen Scientific (USA). Primary antibodies against β-actin (ab8226), pan Cytokeratin (ab217916), Ezrin (ab133297), p-Ezrin (ab52916), Podocalyxin (PODXL, ab269888), Src (ab109381), p-Src (ab32078), JNK (ab76125), *p*-JNK (ab307802), paxillin (ab32084) and p-paxillin (ab76235) were purchased from Abcam (Shanghai, China).

### Cell lines and cell culture

Human breast cancer cell lines MDA-MB-231, MDA-MB-468, and MCF-7 were purchased from the American Type Culture Collection (Manassas, VA, USA). These human breast cancer cell lines were cultured in Dulbecco's Modified Eagle Medium (DMEM) supplemented with 10% fetal bovine serum, penicillin (100 U/mL), and streptomycin (100 mg/L), and maintained in a 37 °C incubator with 5% CO_2_. The cells were passaged every 3–4 d. All experiments were performed using cells within 30 passages, and each experiment was repeated at least three times independently.

### Animal studies and ethics statement

Female BALB/c Nu nude mice, weighing between 18 and 22 g, were obtained from Vital River Laboratory Animal Technology Co., Ltd. (Beijing, China). All procedures involving animal use were conducted in strict accordance with the protocol approved by the Animal Ethics Committee of Jiangxi University of Chinese Medicine (Approval number: 20220218002). Cells were suspended at a concentration of 5 × 10^7^ cells/mL, and a 0.1 mL aliquot of this suspension (5 × 10^6^ cells) was subcutaneously inoculated under sterile conditions into the second pair of mammary glands on the left side of BALB/c Nu nude mice to establish an orthotopic transplantation tumor model. Once the tumor reached approximately 100 mm^3^ in volume, the mice were randomly assigned to treatment groups (*n* = 6). The experiment concluded when the tumor diameter exceeded 20 mm or tumor growth had persisted for 12 weeks. The mice were euthanized by terminal cardiac puncture under deep isoflurane anesthesia (5% for induction and 2% for maintenance) via inhalation, and death was confirmed by the absence of respiratory movements and loss of corneal reflex.

### Circulating tumor cells (CTC) capture and identification

At the experiment endpoint, the mice were euthanized, and an average of 1 mL of blood was collected via cardiac puncture using EDTA vacutainers. Within 1 h of drawing, the blood was lysed in isotonic ammonium chloride buffer (155 mM NH_4_Cl, 10 mM KHCO_3_, 0.1 mM EDTA, pH 7.4) at room temperature (15 °C–25 °C) for 5 min. Following centrifugation, the cell pellet was washed, resuspended in PBS, and adhered to Premium Adhesion Slides (VWR, Indianapolis, Indiana). The cells were then incubated at 37 °C for 20 min to promote attachment. Subsequently, the cells were fixed with 2% paraformaldehyde (pH 7.2–7.4) for 20 min, washed twice with PBS, and blocked with 20% human AB serum (GeminiBio, West Sacramento, CA) in PBS at 37 °C for an additional 20 min. The slides were then incubated with a monoclonal pan-cytokeratin antibody (1:100 dilution) at 37 °C for 1 h. After washing twice with PBS, the slides were incubated with an AF488-labeled secondary antibody (1:500 dilution) at 37 °C for 30 min. Nuclei were counterstained with DAPI at room temperature for 20 min. Finally, the slides were mounted using an aqueous mounting medium (20 mM Tris, pH 8.0, 0.5% n-propyl gallate, and 90% glycerol). Finally, the CTCs were counted, and images were captured using a fluorescence inverted microscope.

### Nest PCR and qualitative real-time PCR (qRT–PCR)

The mice were euthanized after drug administration, and lung tissues were aseptically dissected. The tissues were subsequently rinsed with RNA-free and sterile physiological saline, followed by total RNA extraction using the RNeasy mini kit. Human *cytokeratin 19* (*ck19*) mRNA was amplified by nested PCR using the OneStep Ahead PCR kit to assess the presence of human *ck19* in mouse lung tissues and evaluate breast cancer cell lung metastasis. The methodology, procedural steps, and amplification conditions employed for nested PCR are detailed in Wang et al.^17^ Briefly, after reverse transcription, a first‑round PCR was performed using outer primers. An aliquot of the first‑round PCR products was used as a template for a second‑round conventional PCR with inner primers; the second‑round products were then subjected to agarose gel electrophoresis and visualized using an EC3 gel imaging system. Band analysis was performed using ImageProPlus 5.0 software. Another aliquot of the first‑round PCR products was used directly as a template for real‑time quantitative PCR (qPCR) with inner primers to quantify the relative expression levels of *ck19* mRNA. Results were normalized against β-actin, and relative expression levels of mRNA were calculated using the 2^*−*ΔΔ*Cq*^ method.

### Cell vertical migration assay

Cell vertical migration experiments were performed using the AP48 Chamber (Neuro Probe Inc., Gaithersburg, MD). A total of 30 μL of serum-free DMEM containing 0.1% BSA was added to the lower wells of the Chamber system. A 100  μL cell suspension containing 2 × 10^5^ cells resuspended in serum-free DMEM supplemented with 0.1% BSA was introduced into the upper wells and incubated at 37 °C in a humidified atmosphere containing 5% CO_2_ for 24 h. Following incubation, the filter membrane was detached and fixed with methanol for 10 min. The cells were then stained with a 50 g/L crystal violet solution for 30 min. The number of migratory cells was quantified across five distinct fields of view per well, and an average value was calculated. Three wells were established in parallel for each experimental group.

### Cell horizontal migration

Cell horizontal migration was performed using the Oris™ cell migration system. Cells in the logarithmic growth phase were digested and resuspended to a final density of 3 × 10^5^ cells/mL. Subsequently, 100 μL of this suspension was seeded into each well of a Collagen I-coated Oris™ Pro plate and incubated at 37 °C in a 5% CO_2_ atmosphere for 24 h. Then, the stoppers (cell seeding tools used to generate a central cell-free zone) were removed, and the culture medium was replaced with complete cell culture medium supplemented with various treatments for further cultivation. Following incubation, the medium was discarded, and wells were washed twice with PBS. Next, the cells were stained with Calcein AM (final concentration 2 μM) for 30 min.^18^ Fluorescence inverted microscopy was employed to capture images.

### F-actin staining

Cells in the logarithmic growth phase were harvested and resuspended at a density of 2 × 10^6^ cells/mL. Two hundred microliters of the cell suspension were added to each well of an 8-well ChamberSlide system (Nunc™, USA). The cells were then incubated at 37 °C for 24 h. Subsequently, the culture medium was replaced with complete cell culture medium containing PDS-0330 (30 μM) or vehicle control, and the cells were further cultured for an additional 24 h. Upon completion of the culture period, the chamber was removed, and AlexaFluor488® Phalloidin (1:800 dilution) was applied to the slide. The sample was then incubated in the dark for 15 min. Finally, the arrangement of cytoskeletal F-actin was observed and documented using a fluorescence inverted microscope.

### Cell immunofluorescence

Cells in the logarithmic growth phase were seeded into 16-well ChamberSlide culture plates and incubated for 24 h. Following incubation, the culture medium was replaced with media containing various treatments for an additional 24 h of culture. After washing with PBS and blocking with BSA (4%), a primary antibody dilution (1:200) was added, and the cells were incubated overnight at 4 °C. Upon completion of the incubation, the cells were washed again, and the secondary antibody was diluted 1:300 in the dark; 50 μL of antibody was added to each well, and the plates were gently mixed on a shaker for 2 h in the dark. Subsequently, the slides were incubated with an appropriate volume (100 or 200 μL) of 4′,6-diamidino-2-phenylindole (DAPI) staining solution for 10 min and subsequently washed three times. Finally, images were captured under an inverted fluorescence microscope.

### Immunohistochemical analysis

Immunohistochemical analysis was performed to assess protein expression in breast cancer tissues. The specific procedures were as follows. The tumor tissues were fixed (with 4% Paraformaldehyde Fix Solution), dehydrated, and subsequently embedded in paraffin blocks. Continuous 4 μm-thick sections were then cut and baked at 65 °C for 30 min. These sections were placed into the BenchMark XT system for fully automated immunohistochemical staining. The staining protocol included baking at 75 °C for 4 min; dewaxing using EZ Prep solution (Ventana Medical Systems; Marana, AZ) at 76 °C for 4 min; antigen retrieval with Cell Conditioning Solution at 99 °C for 30 min; incubation with proteinase K inhibitor for an additional 30 min; incubation with antibody at 37 °C for 30 min; washing with Reaction Buffer Concentration (10×) for 5 min; and incubation with a secondary antibody at 37 °C for 8 min. Peroxidase activity was then detected using the SignalStain DAB Substrate kit (Cell Signaling Technology) or AEC Substrate system (ab64252; Abcam). Typical images were captured under a microscope (BX63, Olympus).

### Immunoblotting assay

After the treatment, the cells were collected, washed, and lysed (RIPA Lysis and Extraction Buffer). The total protein content was quantified using the Coomassie brilliant blue method. Equal concentrations of protein (20–40 μg of total proteins) were subjected to electrophoresis on an SDS–polyacrylamide gel. Subsequently, the membrane was transferred, blocked (4% BSA), and incubated overnight at 4 °C with a primary antibody (1:1000 dilution). The membrane was then washed with Tween Tris buffer saline solution (TTBS) and incubated with a secondary antibody (1:1000 dilution) at room temperature for 2 h. After another round of washing, the membrane was developed with 3,3′-diaminobenzidine (DAB) as a chromogenic agent. Images were captured utilizing the EC3 gel imaging system. Band analysis was performed using ImageJ software, and the relative expression level of the protein was expressed as the ratio of the target band to that of an internal reference band.

### Co-immunoprecipitation

Cells were harvested following treatments and lysed on ice for 45 min using a co-immunoprecipitation protein lysis buffer provided in the Pierce™ Co-Immunoprecipitation Kit (Thermo Scientific™). The lysate was then centrifuged at 12,000 × *g* for 30 min. The supernatant was collected, and either 1 μg of anti-Ezrin or anti-PODXL antibody was added to the cell lysate, along with 25 μL of protein A/G beads. This mixture was incubated overnight at 4 °C with gentle shaking to facilitate immunoprecipitation. Subsequently, the mixture was centrifuged at 3,000 × *g* for 5 min at 4 °C, allowing the protein A/G beads to pellet at the bottom of the tube. The supernatant was carefully removed, and the protein A/G beads were washed with 1 mL of lysis buffer. Finally, 15 μL of a 2 × SDS loading buffer was added to the sample, which was then boiled for 10 min. The samples separated via SDS–PAGE and analyzed by Western blotting.

### Cell transfection

Logarithmic-growth-phase MDA-MB-468 and MCF-7 cells infected with lentiviruses establish two stably transfected cell lines: MDA-MB-468/CLDN1-sh and MCF-7/CLDN1. The cells were seeded at a density of 5 × 10^5^ per well in 6-well plates and cultured overnight. Subsequently, LV-CLDN1 shRNA (CLDN1 Human shRNA Lentiviral Particle, Locus ID 9076) or LV-CLDN1 (CLDN1 Human Tagged ORF Clone Lentiviral Particle, NM_021101) was added and thoroughly mixed with the cells. The mixtures were then incubated in a humidified incubator at 37 °C with 5% CO_2_ for 8 h. Subsequently, the medium was replaced with complete DMEM. The cells were harvested after an additional 48 h, and the infection efficiency was assessed by qPCR analysis. A concentration of 2 mg/L puromycin was employed for screening purposes to establish stable infected cell lines. The negative control was transfected with LV‑NC lentivirus using the same method.

### RNA interference

RNA interference was used to silence the *ezrin* gene expression in MCF-7 cells stably transfected with the *cldn1* gene, per our previously established methodology.^19^ MCF-7/CLDN1 cells in the logarithmic growth phase were seeded at a density of 5 × 10^5^ cells per well in 6-well plates and cultured overnight. Subsequently, SignalSilence Ezrin siRNA I (Cell Signaling Technology, Inc.; Cat. No: 6475) or SignalSilence Control siRNA (Cell Signaling Technology, Inc.; Cat. No: 6568) was introduced and co-cultured for a duration of 24 h. The modified MCF-7 cells were utilized for subsequent experiments.

### Statistical analysis

Statistical analyses were conducted using SPSS 13.0 statistical software. The results are expressed as mean ± standard deviation (SD). Comparisons between two groups were analyzed using Student's *t*‑test. For comparisons involving three or more groups, one‑way analysis of variance (ANOVA) was used, followed by Fisher's LSD test as the post‑hoc test. A *p*-value < 0.05 was considered to indicate significance.

## Results

### Pharmacological inhibition of CLDN1 impairs migration in CLDN1-high TNBC cells

To evaluate the role of CLDN1 in TNBC, we first assessed its protein expression in the TNBC cell lines MDA-MB-468 and MDA-MB-231. Western blot analysis confirmed that CLDN1 expression was significantly higher in MDA-MB-468 cells than in MDA-MB-231 cells (Figure 1a). We next treated both cell lines with PDS‑0330, a small-molecule inhibitor of CLDN1, and evaluated its effects on migration using two complementary assays. In the AP48 chamber system, PDS-0330 (30 μM) significantly reduced the number of migrated MDA-MB-468 cells (Figure 1b). Similarly, in the Oris migration assay, PDS-0330 (30 μM) treatments markedly inhibited horizontal migration of MDA-MB-468 cells, as indicated by the enlarged central cell-free area and reduced fluorescence intensity. In contrast, no significant changes were observed in MDA-MB-231 cells (Figure 1c).

**Figure 1.**
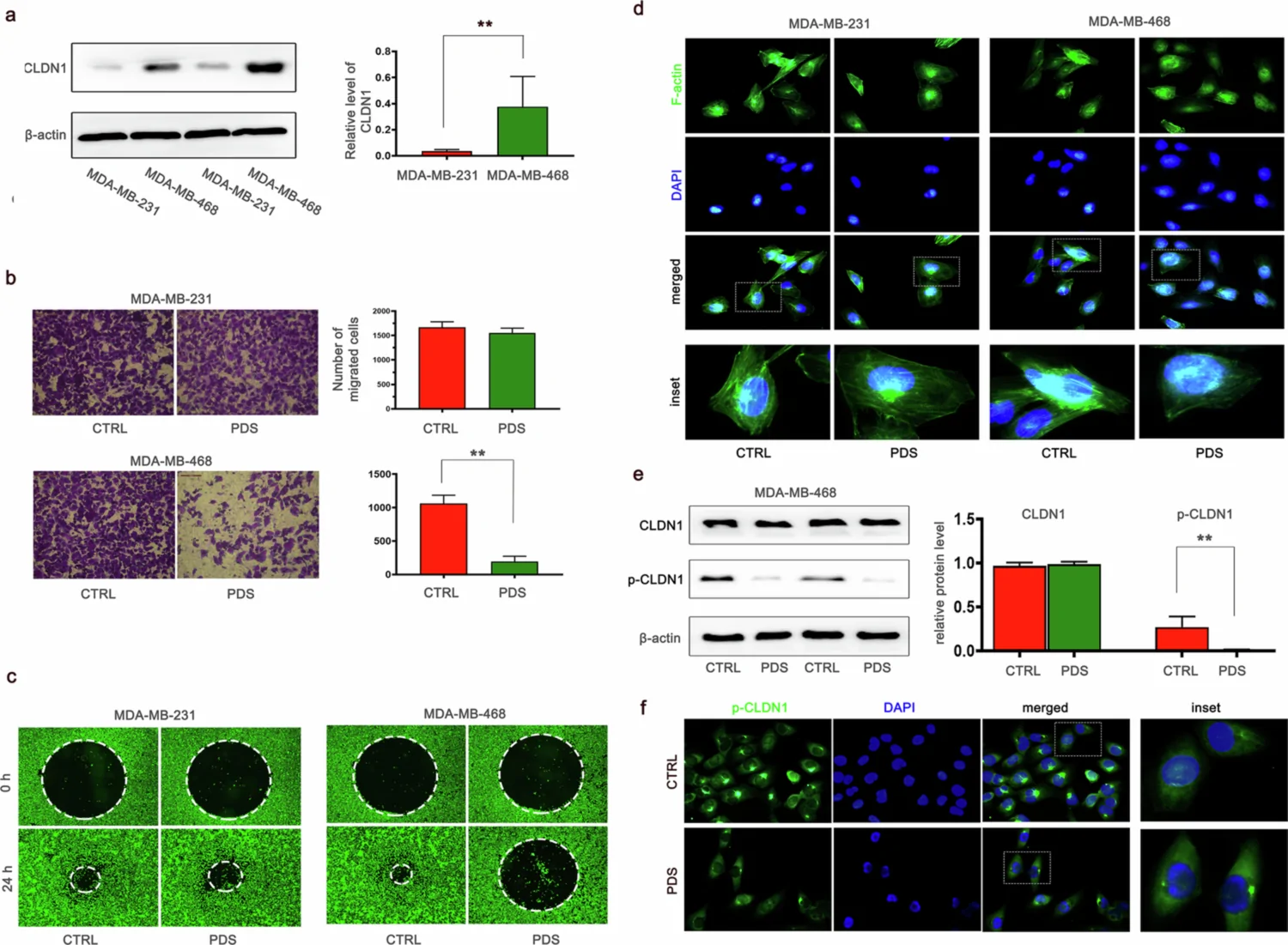
Pharmacologically targeting CLDN1 impairs cell migration in TNBC. (a) MDA-MB-468 cells exhibited significantly higher levels of CLDN1 expression compared to MDA-MB-231 cells; (b, c) PDS-0330 inhibits cell vertical (b) and horizontal (c) migration in MDA-MB-468 cell, but no effect on that in MDA-MB-231; (d) PDS-0330 impairs F-actin organization in MDA-MB-468 cells; (e, f) PDS-0330 inhibited the phosphorylation of CLDN1, but no changes in total CLDN1 expression were observed in Western blot assays (e) and immunofluorescence assays (f). All experiments were performed independently three times (*n* = 3). Data are presented as mean ± SD. Statistical significance was determined by Student's *t*-test. **p < 0.01. CLDN1; Claudin-1; CTRL: control; PDS: PDS-0330 (30 μM for 24 h).

Because cell migration often involves cytoskeletal reorganization, we examined F‑actin organization by Phalloidin staining. In the control MDA-MB-468 cells, F-actin formed well-aligned filaments with enriched cortical staining. Treatment with PDS-0330 disrupted this organization, leading to cell shrinkage and disorganized F‑actin structures. Again, MDA-MB-231 cells showed no notable alterations (Figure 1d). We further confirmed that PDS-0330 reduced p-CLDN1 levels while total CLDN1 levels remained unchanged, as shown by Western blotting and immunofluorescence (Figure 1e and f). Immunofluorescence analysis showed that phosphorylation of CLDN1 exhibited cortical localization in control cells, which was notably diminished following PDS-0330 treatment (Figure 1f).

### CLDN1 promotes migration and metastasis *in vitro* and *in vivo*

To further establish the functional role of CLDN1, we genetically modulated its expression in TNBC cells (MDA-MB-468) and in a complementary gain-of-function model using MCF-7 cells, which have low endogenous CLDN1 expression. Knockdown of *cldn1* in MDA-MB-468 cells (MDA‑MB-468/CLDN1-sh) substantially reduced total CLDN1 and p-CLDN1 levels. In contrast, for overexpression studies, we chose MCF-7 cell, a breast cancer cell line exhibits very low endogenous CLDN1 expression (as shown in Figure 2a), making it suitable for evaluating gain-of-function effects. Overexpression of CLDN1 in MCF-7 cells (MCF-7/CLDN1) increased p-CLDN1 levels (Figure 2a). Functionally, *cldn1* knockdown strongly suppressed cell migration; in contrast, its overexpression enhanced migration capacity (Figure 2b). Consistent with pharmacological inhibition, *cldn1* knockdown disrupted F-actin architecture, while *cldn1* overexpression promoted more organized actin filaments (Figure 2c). These data suggest that CLDN1 is involved in migration and actin organization. The MCF-7 results cannot directly confirm CLDN1 function in TNBC because MCF-7 is an ERα-positive breast cancer cell line. However, given its low endogenous CLDN1 expression, these data provide supportive gain-of-function evidence for a role of CLDN1.

**Figure 2.**
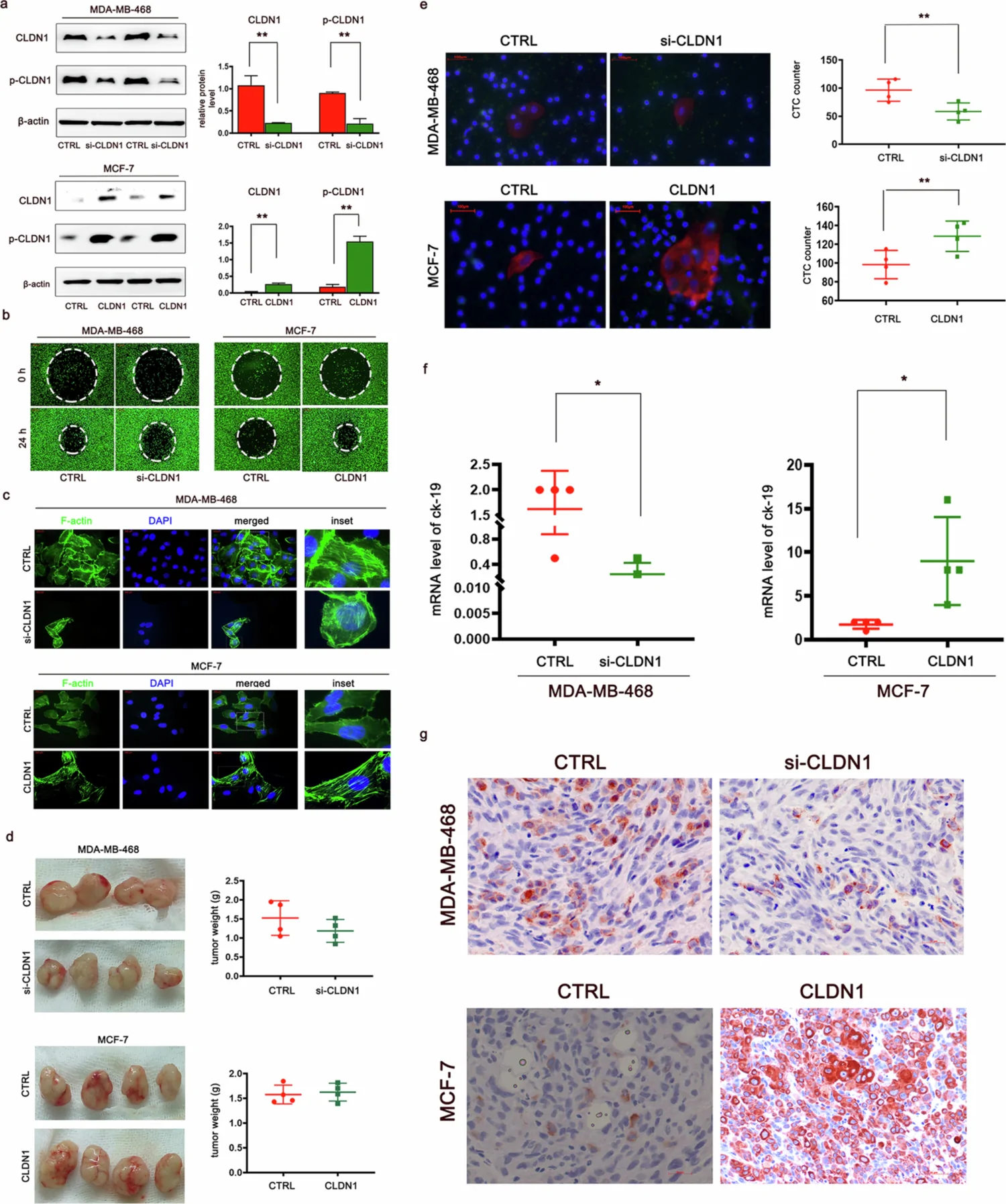
CLDN1 promotes cell migration and metastasis in breast cancer. (a) Expression of CLDN1 and *p*-CLDN1 in MDA-MB-468/CLDN1-sh and MCF-7/CLDN1 cells; (b) Horizontal migration of MDA-MB-468/CLDN1-sh cells and MCF-7/CLDN1; (c) F-actin organization in MDA-MB-468/CLDN1-sh cells and MCF-7/CLDN1. (d) tumor weight in MDA-MB-468/CLDN1-sh and MCF-7/CLDN1-bearing mice; (e) CTC counts in MDA-MB-468/CLDN1-sh and MCF-7/CLDN1-bearing mice; (f) human *ck19* gene expression in lung tissues in MDA-MB-468/CLDN1-sh and MCF-7/CLDN1-bearing mice; (g) p-CLDN1 expression in tumor blocks of mice bearing MDA-MB-468/CLDN1-sh and MCF-7/CLDN1 tumors. All experiments were performed independently three times (*n* = 3). Data are presented as mean ± SD. Statistical significance was determined by Student's *t*-test. *p < 0.05, **p < 0.01. CLDN1: Claudin-1; CTRL: control.

We then extended these findings to an *in vivo* setting. In the mouse model, neither *cldn1* knockdown nor overexpression affected primary tumor growth (Figure 2d). But cldn1 knockdown significantly reduced the number of CTCs and *ck-19* mRNA levels in lung tissue, which is consistent with a potential impairment of hematogenous dissemination. Conversely, *cldn1* overexpression elevated CTC counts and lung *ck-19* expression (Figure 2e and f). These data suggest that alterations in CLDN1 expression were associated with altered hematogenous dissemination, without an apparent effect on tumor proliferation. An immunohistochemical analysis of tumor sections further confirmed that p-CLDN1 levels decreased upon CLDN1 knock-down and increased upon its overexpression (Figure 2g), confirming that CLDN1 activity modulates hematogenous dissemination *in vivo*.

### Ezrin is involved in CLDN1-stimulated cell migration

Ezrin serves as a critical node linking extracellular signals to F-actin dynamics and cytoskeletal remodeling. To investigate whether Ezrin is involved in the F-actin arrangement and migration stimulated by CLDN1, we first assessed the effects of PDS-0330 on Ezrin expression and binding activity. PDS-0330 (30 μM) significantly inhibited Ezrin phosphorylation but had no notable effect on its total expression in MDA-MB-468 cells (Figure 3a). In our co-immunoprecipitation assays, using an anti-Ezrin antibody as the precipitating agent resulted PODXL content reduction in co-sediments; however, no significant alteration in Ezrin levels was observed. In contrast, when PODXL antibody was employed as the precipitating agent, PDS-0330 reduced both Ezrin and PODXL contents in the co-sediments (Figure 3b). These findings indicate that PDS-0330 disrupts Ezrin protein phosphorylation in MDA-MB-468 cells, consequently influencing its interaction with PODXL.

**Figure 3.**
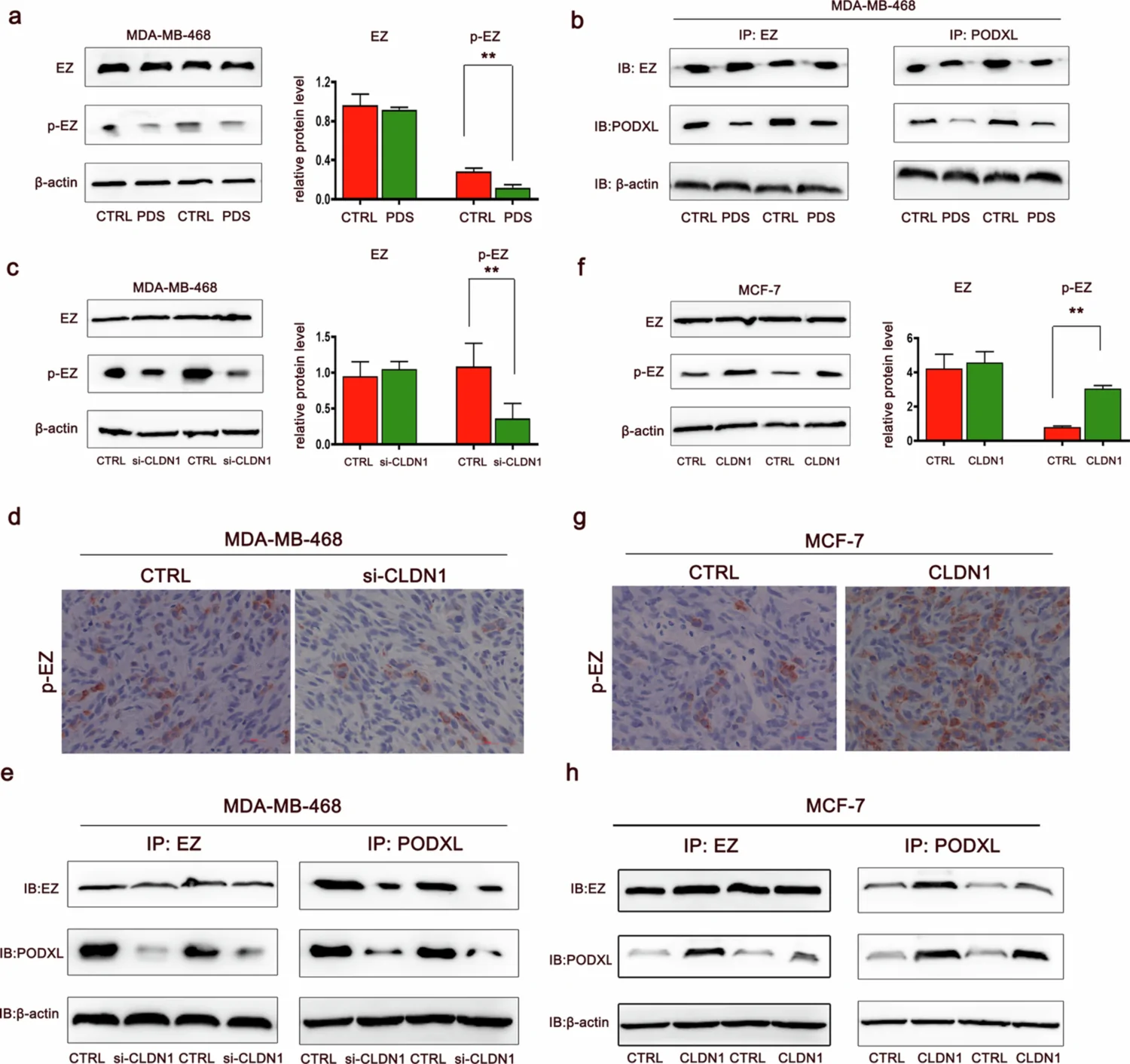
CLDN1 promotes Ezrin activation in MDA-MB-468/CLDN1-sh and MCF-7/CLDN1 cells. (a, b) PDS-0330 inhibits Ezrin phosphorylation (a) and disrupts the interaction between Ezrin and PODXL (b), while exhibiting no notable effect on total Ezrin expression in MDA-MB-468 cells; (c–e) Inhibitory effects of *cldn1* gene knock-down on p-Ezrin expression *in vitro* (c) and *in vivo* (d) as well as the interaction between Ezrin and PODXL in MDA-MB-468/CLDN1-sh cells (e); (f–h) up-regulatory effects of *cldn1* gene knock-in on p-Ezrin expression *in vitro* (f) and *in vivo* (g), as well as the interaction between Ezrin and PODXL in MCF-7/CLDN1 cells (h). All experiments were performed independently three times (*n* = 3). Data are presented as mean ± SD. Statistical significance was determined by Student's *t*-test. **p < 0.01. CLDN1; Claudin-1; CTRL: control; EZ: Ezrin; IB: immunoblot; IP: immunoprecipitation; PDS: PDS-0330 (30 μM for 24 h); PODXL: podocalyxin.

In MDA-MB-468 cells, *cldn1* knockdown significantly reduced p-Ezrin level; however, no notable changes were observed in total expression (Figure 3c). Similarly, the inhibitory effects of *cldn1* knock-down on p-Ezrin level were also detected in tumor blocks from nude mice (Figure 3d). Furthermore, *cldn1* knock-down impaired the Ezrin–PODXL interaction in MDA-MB-468/CLDN1-sh cells (Figure 3e). In contrast, in MCF-7 cells, the introduction of *cldn1* resulted in a significant increase phosphorylation of Ezrin within cellular and tumor tissues (Figure 3f and g), along with enhanced Ezrin–PODXL interactions (Figure 3h). These findings strongly support an association between CLDN1 status and Ezrin phosphorylation/activity across the gain- and loss-of-function models.

Furthermore, the *ezrin* gene was knocked down in MCF-7/CLDN1 cells. Following the introduction of *cldn1*, p-Ezrin and p-CLDN1 levels increased significantly compared with the vector control; however, total Ezrin expression remained unchanged. Conversely, after knocking down the *ezrin* gene, both Ezrin and p-Ezrin level were significantly reduced, with no notable changes in phosphorylation of CLDN1 (Figure 4a). Co-IP assays revealed that *ezrin* gene knock-down disrupted the CLDN1-induced Ezrin–PODXL interaction in MCF-7/CLDN1 cells (Figure 4b). Similarly, the MCF-7/CLDN1 cells' migratory capacity and F-actin organization were significantly diminished following *ezrin* knock-down (Figure 4c and d). Together, these findings support an association between CLDN1 status and Ezrin activity, with the MCF-7 results providing supportive gain-of-function evidence rather than direct validation in TNBC.

**Figure 4.**
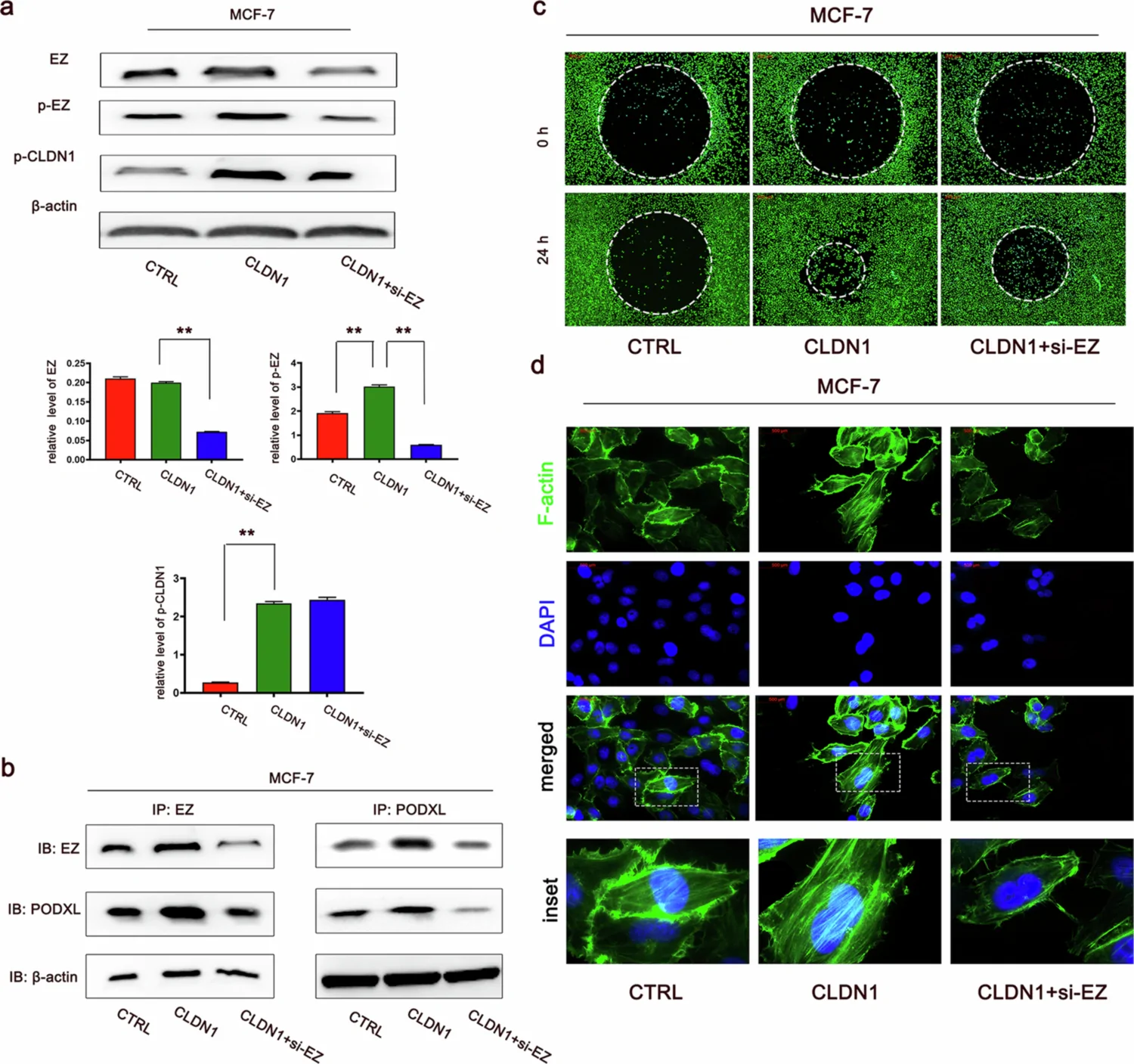
Effects of Ezrin gene knock-down on Ezrin activation and migration in MCF-7/CLDN1 cells. (a, b) Effects of *ezrin* gene knock-down on Ezrin, p-Ezrin and p-CLDN1 expression (a) and the interaction between Ezrin and PODXL in MCF-7/CLDN1 cells (b); (c, d) Effects of *ezrin* gene knock-down on migration (c) and F-actin organization (d) in MCF-7/CLDN1 cells. All experiments were performed independently three times (*n* = 3). Data are presented as mean ± SD. Statistical analysis was performed using one-way ANOVA followed by Fisher's LSD test for multiple comparisons. **p < 0.01. CLDN1; Claudin-1; CTRL: control; EZ: Ezrin; IB: immunoblot; IP: immunoprecipitation; PDS: PDS-0330 (30 μM for 24 h); PODXL: podocalyxin.

### CLDN1 promotes Src signal pathway activation

We assessed the expression of Src and its downstream targets, JNK and paxillin, to determine whether Src kinase is involved in CLDN1-mediated effects on cell motility. Western blotting demonstrated that PDS-0330 markedly inhibited the phosphorylation of Src kinase in MDA‑MB‑468 cells, along with that of its downstream targets, JNK and paxillin, both which are associated with cellular movement and migration (Figure 5a). Similarly, MDA-MB-468/CLDN1-sh cells exhibited a substantial decrease in phosphorylated Src, JNK, and paxillin (Figure 5b). In MCF-7/CLDN1 cells, p-Src, p-JNK, and p-paxillin demonstrated marked increases (Figure 5c). Moreover, in MCF-7/CLDN1, knock-down of *ezrin* gene did not result in a significant impact on the expression levels of Src, p-Src, p-JNK, and p-paxillin (Figure 5d). To specifically inhibit Src family kinase activity, we chose AZD0530, a potent and selective Src inhibitor widely used in mechanistic studies of Src-dependent signaling. As shown in Figure 5b–d, AZD0530 demonstrated a substantial inhibitory effect on Src kinase activity. Moreover, AZD0530 treatment significantly reduced the migration of MCF-7/CLDN1 cells, as shown in Supplementary Figure S1. Taken together, these findings support an association between CLDN1 status, Src pathway activation, and migration, and are consistent with a model in which CLDN1 promotes migration via Src-associated signaling and downstream Ezrin activation.

**Figure 5.**
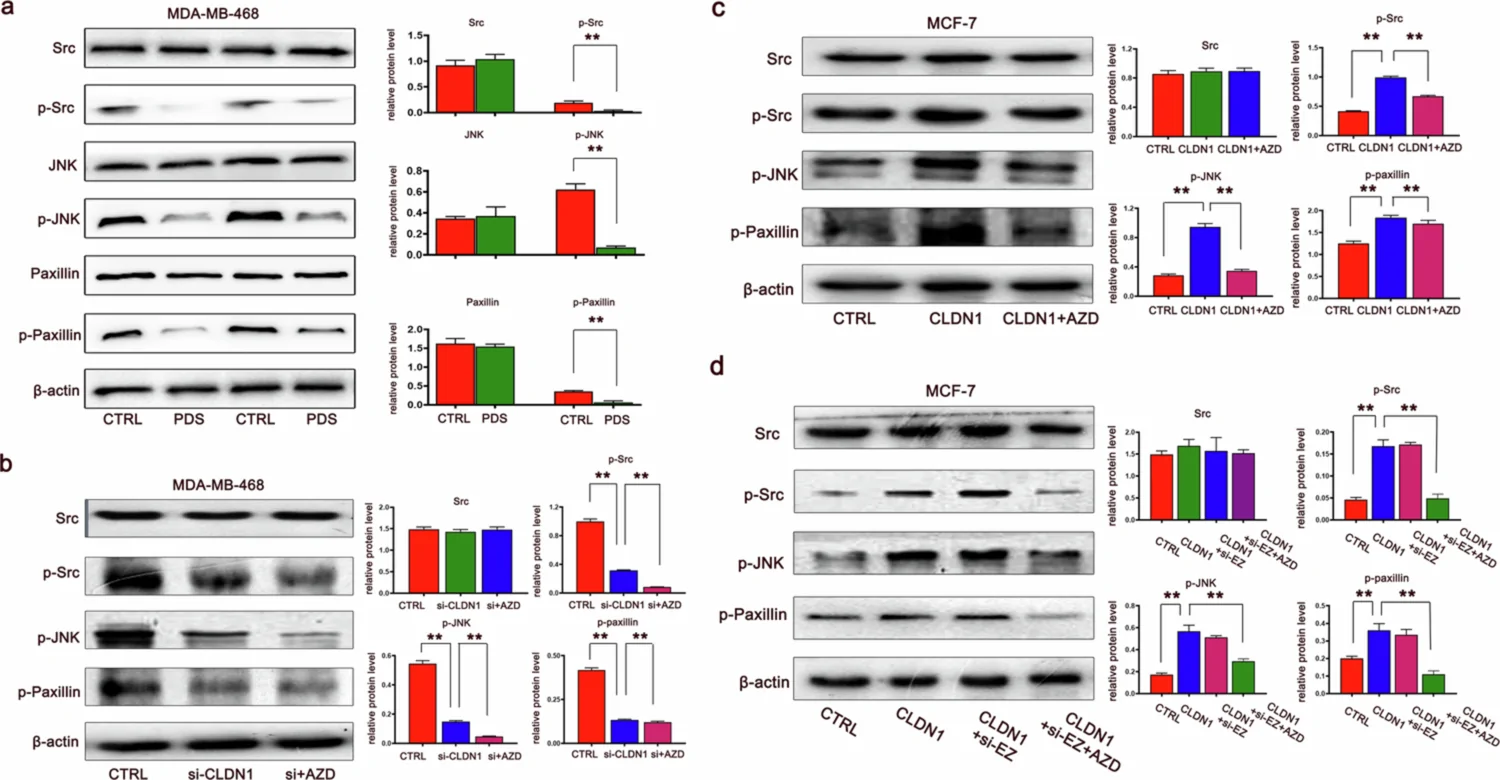
CLDN1 promotes the activation of Src signal pathway. (a) PDS-0330 inhibits the phosphorylation of Src kinase, as well as the phosphorylation of its downstream targets JNK and paxillin in MDA-MB-468 cells; (b) Effects of *cldn1* gene knock-down on expression of p-Src and its downstream targets JNK and paxillin in MDA-MB-468/CLDN1-sh cells; (c) Effects of *cldn1* gene knockin on expression of p-Src and its downstream targets JNK and paxillin in MCF-7/CLDN1 cells; (d) Effects of knock-down of the *ezrin* gene on the expression levels of Src, p-Src, p-JNK, and p-paxillin. All experiments were performed independently three times (*n* = 3). Data are presented as mean ± SD. Statistical significance for two-group comparisons (a) was determined by Student's *t*-test. For multi-group comparisons (b–d), one-way ANOVA followed by Fisher's LSD test was used. **p < 0.01. AZD: AZD0530 (15 μM for 24 h); CLDN; Claudin-1; CTRL: control; EZ: Ezrin; JNK: c-Jun N-terminal kinase; PDS: PDS-0330 (30 μM for 24 h).

## Discussion

Breast cancer is the second leading cause of cancer-related deaths in women. In 2020, Breast cancer caused approximately 685,000 global deaths.^20^ As a subtype of breast cancer, TNBC, accounting for 10% to 20% of all breast cancers, shows more aggressive clinical features than others. Consequently, it is urgent to identify novel and positive therapeutic targets that can effectively address the current limitations in treatment strategies. CLDN1 is a crucial member of the tight junction protein family and serves as one of the primary components of tight junctions.^8^ Researches have demonstrated that CLDN1 plays a significant role in the malignant progression of several cancers. In addition, the survival analysis derived from the Kaplan-Meier Plotter database (http://kmplot.com/analysis/) indicates that patients with TNBC exhibiting high expression of CLDN1 have significantly shorter Q3 survival times. Although the overall survival differences did not reach statistical significance, this finding suggests a potential trend indicating that high CLDN1 expression may be associated with poor prognosis in CLDN1-overexpressing TNBC patients (refer to Supplementary data for detailed results, Figure S2). However, in breast cancer, the expression of CLDN1 is rather complex, with significant differences in expression levels across different subtypes of breast cancer. Recent studies have demonstrated that in over 80% of cases of triple-negative breast cancer (TNBC), CLDN1 displays abnormal expression patterns, characterized by elevated levels and atypical localization. However, the relationship between this aberrant expression pattern and the clinical outcomes of TNBC remains to be further explored.

Guided by these concepts, this study focused on the effects of CLDN1 on the migration and metastasis of TNBC using two distinct TNBC cell lines: MDA-MB-231 and MDA-MB-468. These cell lines represent different subtypes of TNBC and exhibit varying expression patterns of CLDN1, as indicated by data from the Human Protein Atlas (HPA). In MDA-MB-231 cells, which display low levels of CLDN1 expression, exposure to the CLDN1 inhibitor PDS-0330 did not result in significant changes in cell migration. In contrast, MDA-MB-468 cells, characterized by high expression levels of CLDN1, showed a marked reduction in migratory capacity upon treatment with PDS-0330. Furthermore, PDS-0330 significantly influenced both the expression and organization of F-actin within MDA-MB-468 cells, which is the critical modulator of cell migration.^21^ Moreover, it was observed that PDS-0330 significantly reduced the expression of phosphorylated CLDN1 (p-CLDN1), but it did not affect total CLDN1 levels. The results obtained from both knock-down and knock-in experiments further substantiated that CLDN1 plays a critical role in influencing the migratory behavior, CTC counts, and metastatic potential of the high-CLDN1 TNBC. These findings suggest, in this high-CLDN1 TNBC model, migratory and metastatic capacities are associated with CLDN1 expression status.

Ezrin, a member of the ERM (Ezrin/Radixin/Moesin) family, was originally identified as an actin-binding protein within the microvilli structure, playing a crucial role in cell motility.^22^ As a substrate for tyrosine kinases, Ezrin serves as crosslinker between receptors, ion channels, transporters and cortical actin filaments through scaffold proteins.^23^ PODXL (Podocalyxin), a type 1 sialomucin that belongs to the CD34 family, is a crucial scaffold protein. PODXL directly interacts with the FERM domain at the N-terminus of the Ezrin protein via its C-terminal PDZ binding region (DTHL domain).^24^ This kind of interaction plays a significant role in mediating tumor cell migration and invasion. Furthermore, Takasawa's research (2021) has confirmed that the expression of CLDN1 not only impacts the function of tight junctions but also modulates the expression of a range of proteins associated with cell adhesion and actin cytoskeleton remodeling, including Ezrin/Radixin/Moesin.^25^

Our previous findings demonstrated the role of CLDN1 on the migration in CLDN1 highly expressive cells, and this effect may be associated with the disruption of F-actin arrangement. Therefore, to investigate whether Ezrin is involved in these effects, the impact of CLDN1 on Ezrin expression and binding activity were assessed. The results indicated that PDS-0330 effectively inhibits Ezrin phosphorylation and destroys the binding activity with PODXL in MDA-MB-468 cells. Moreover, both knock-down and knock-in experiments confirmed that the activation of Ezrin is modulated by CLDN1. Additionally, small RNA targeting *ezrin* was introduced into MCF-7 cells overexpressing CLDN1 to silence the *ezrin* gene. The results revealed that silencing the *ezrin* gene not only inhibited Ezrin phosphorylation but also affected its interaction with PODXL, as well as cell migration and F-actin arrangement. Furthermore, when *cldn1* was knocked down or knocked in, changes in Ezrin expression corresponded to variations in CLDN1 expression status. However, when *ezrin* was knocked down, no significant changes were noted in p-CLDN1 levels. These results indicate that within the regulatory framework governing cell migration induced by CLDN1 in CLDN1-overexpressing TNBC, Ezrin may serve as one of downstream targets of CLDN1.

As a substrate for kinases, the phosphorylation of Ezrin by various signaling mechanisms represents a primary regulatory mode of Ezrin activity.^26^ ^,^ ^27^ To investigate whether kinase systems are involved in the regulation of CLDN1 on metastasis in TNBC, we explored signaling pathways within the KEGG pathway database that are concurrently associated with both CLDN1 and Ezrin and are implicated in cell migration and invasion processes. Subsequently, the effects of PDS-0330 on the activities of these kinases were assessed by Glo kinase assay system. The results indicated that Src kinase activity was most significantly impacted by PDS-0330 (refer to Supplementary data for detailed results, Figure S3). Subsequent analyses further confirmed that Src kinase plays a role in the migration and metastasis induced by CLDN1. Notably, regardless of whether CLDN1 was knocked down or overexpressed, alterations in CLDN1 status resulted in corresponding changes to the expression levels of p-Src and its downstream targets. Moreover, in MCF-7/CLDN1 cells, silencing the Ezrin gene did not lead to any changes in either the expression or activity of Src and its downstream targets. These findings suggest that while Src activity is influenced by CLDN1 expression status, Ezrin does not appear to affect either the expression or activity of Src.

Taken together, our study indicates that CLDN1 plays a crucial role in regulating the migration and blood-borne metastasis of TNBC with high CLDN1 expression, and this effect may be associated with activation of the Src/Ezrin signaling axis. Targeting CLDN1 presents a promising novel therapeutic strategy for TNBC, particularly for TNBC with high CLDN1 expression, where it may offer superior clinical outcomes.

However, we acknowledge certain limitations within this study. First, only two TNBC cell lines were utilized to investigate the role of CLDN1. A more comprehensive understanding of this topic necessitates validation across a broader range of TNBC cell lines and clinical samples. Second, MCF-7, an ERα-positive and CLDN1-low expression cell line, was employed as parental cells for the CLDN1 gene knock-in instead of MDA-MB-231 cells. This choice was made due to the comparatively weaker migratory capacity of MCF-7 cells relative to MDA-MB-468. Following the knock-in of CLDN1, an enhanced migratory ability was observed in MCF-7 cells; however, MDA-MB-231 cells exhibit strong migratory potential regardless of whether CLDN1 is introduced or not. This may not fully represent the influence of CLDN1 on cell migration. Such a decision could compromise the rigor of our conclusions regarding the mechanisms through which CLDN1 exerts its regulatory effects on TNBCs. Nevertheless, our conclusion that CLDN1 regulates cell migration through the Src/Ezrin signaling axis is not drawn solely from the MCF-7 overexpression model. It is supported by converging evidence from three complementary experimental settings, which consistently demonstrate a positive correlation between CLDN1 expression and Src/Ezrin pathway activation, as well as enhanced migratory capacity.

## Supplementary Material

Supplementary material Fig S2 survival R 9 1.tif

Supplementary material Fig S1 R 9 1.tif

Supplementary material Fig S3 R 9 1.tif

## Data Availability

The analyzed data sets generated during the study are available from the corresponding author upon reasonable request.
